# Graph-based modeling of tandem repeats improves global multiple sequence alignment

**DOI:** 10.1093/nar/gkt628

**Published:** 2013-07-22

**Authors:** Adam M. Szalkowski, Maria Anisimova

**Affiliations:** ^1^Swiss Institute of Bioinformatics, Quartier Sorge Batiment Genopode, 1015 Lausanne, Switzerland and ^2^Department of Computer Science, ETH Zürich, Universitätstrasse 6, 8092 Zürich, Switzerland

## Abstract

Tandem repeats (TRs) are often present in proteins with crucial functions, responsible for resistance, pathogenicity and associated with infectious or neurodegenerative diseases. This motivates numerous studies of TRs and their evolution, requiring accurate multiple sequence alignment. TRs may be lost or inserted at any position of a TR region by replication slippage or recombination, but current methods assume fixed unit boundaries, and yet are of high complexity. We present a new global graph-based alignment method that does not restrict TR unit indels by unit boundaries. TR indels are modeled separately and penalized using the phylogeny-aware alignment algorithm. This ensures enhanced accuracy of reconstructed alignments, disentangling TRs and measuring indel events and rates in a biologically meaningful way. Our method detects not only duplication events but also all changes in TR regions owing to recombination, strand slippage and other events inserting or deleting TR units. We evaluate our method by simulation incorporating TR evolution, by either sampling TRs from a profile hidden Markov model or by mimicking strand slippage with duplications. The new method is illustrated on a family of type III effectors, a pathogenicity determinant in agriculturally important bacteria Ralstonia solanacearum. We show that TR indel rate variation contributes to the diversification of this protein family.

## INTRODUCTION

Today accurate multiple sequence alignment (MSA) is frequently needed in genomics and molecular biology studies. In an alignment defined by the evolution of sequence residues (rather than by its molecular structure), characters in the same column are assumed to be homologous, indicating that they have evolved from a common ancestral character. Recent successful additions to the alignment tools arsenal are the phylogeny-aware algorithms that reduce alignment errors producing biologically more meaningful alignments (1). Graph-based representation of ancestral sequences helps to further reduce error by allowing alternative splicings and tolerating errors in the branching pattern of the guide tree. A similar approach has been applied to next-generation sequences from environmental samples to provide more accurate extensions of reference alignments (2). Recent drive for biologically more meaningful alignments (3) included developments to account for special sequence features such as protein domains, repeats, rearrangements and promoter regions (4–9). Indeed, the global alignment of proteins with shuffled, duplicated, missing or inverted segments may present a substantial challenge. Here, we focus on improving the strategy for aligning sequences with tandem repeats (TRs).

A TR is a consecutive repetition of sequence segments with a similar character pattern. In coding regions, TR mutations have direct effects on the protein product, and even non-coding TRs mutations can seriously impact genetic fitness (10). A number of human proteins with TRs are known to perform important biological functions or to be related to infectious and neurodegenerative diseases. Non-coding TRs in human cells were discovered incidentally, and ever since have been used as biological markers in forensics and genetic profiling (11,12). TRs in genomic sequences may be much more frequent than is typically thought (13,14), covering large parts of proteins (in some cases up to 100%: e.g. collagens that form muscle and connective tissues in animals).

**Figure 1.**
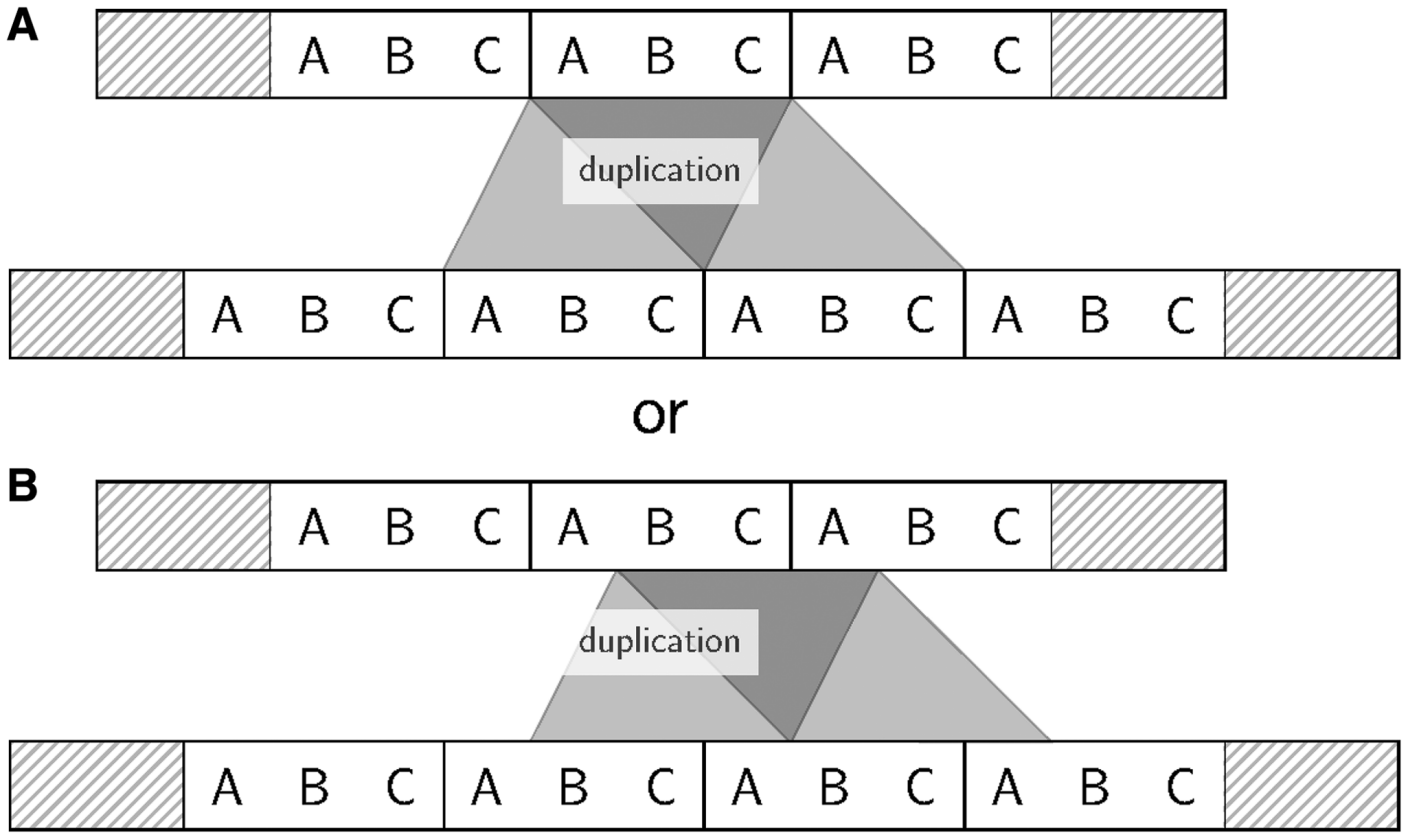
Examples of TR unit duplication: (**A**) in phase and (**B**) not in phase. TR unit deletions or insertions (i.e. duplications) can occur at any position of a TR unit. The structure of the protein is retained independently of the ‘phase’ at which a duplication occurs.

TRs are usually thought to evolve rapidly, but many exceptions of this rule exist (15). Moreover, little is known about the biological processes that shape TRs. TR units may mutate over time or undergo duplications or loss. As time passes, the TR unit similarity fades, making the shared ancestry of multiple repeat units more difficult to detect. Furthermore, accumulating evidence suggests that TRs often mutate by replication slippage (16,17). In sequences with TRs, the mispairing of a slipping-strand during the DNA synthesis may lead to loss or gain of TR units as loops of TR units form hairpin structures (18). The real biological process does not necessarily preserve the ‘phase’ in which TR units are lost or gained. Figure 1 shows an example of slippage mutations starting at different positions of a TR region, yet both resulting in the same protein sequence. As slippage-caused loss or gain of TR units may happen at every position of a TR region, fixed TR unit boundaries are an artificial constraint. When applied to homologous sequences, TR predictors usually will not preserve the phase of TR units (Supplementary Figure S10). Despite this, to ease the modeling and computation, current methods typically assume fixed unit boundaries but are still of high complexity [at least 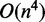 (4,6,7)]. Both our new method and the simulation allow for TR mutations at any position of a TR region and are thus not restricted to unit boundaries.

**Figure 2.**
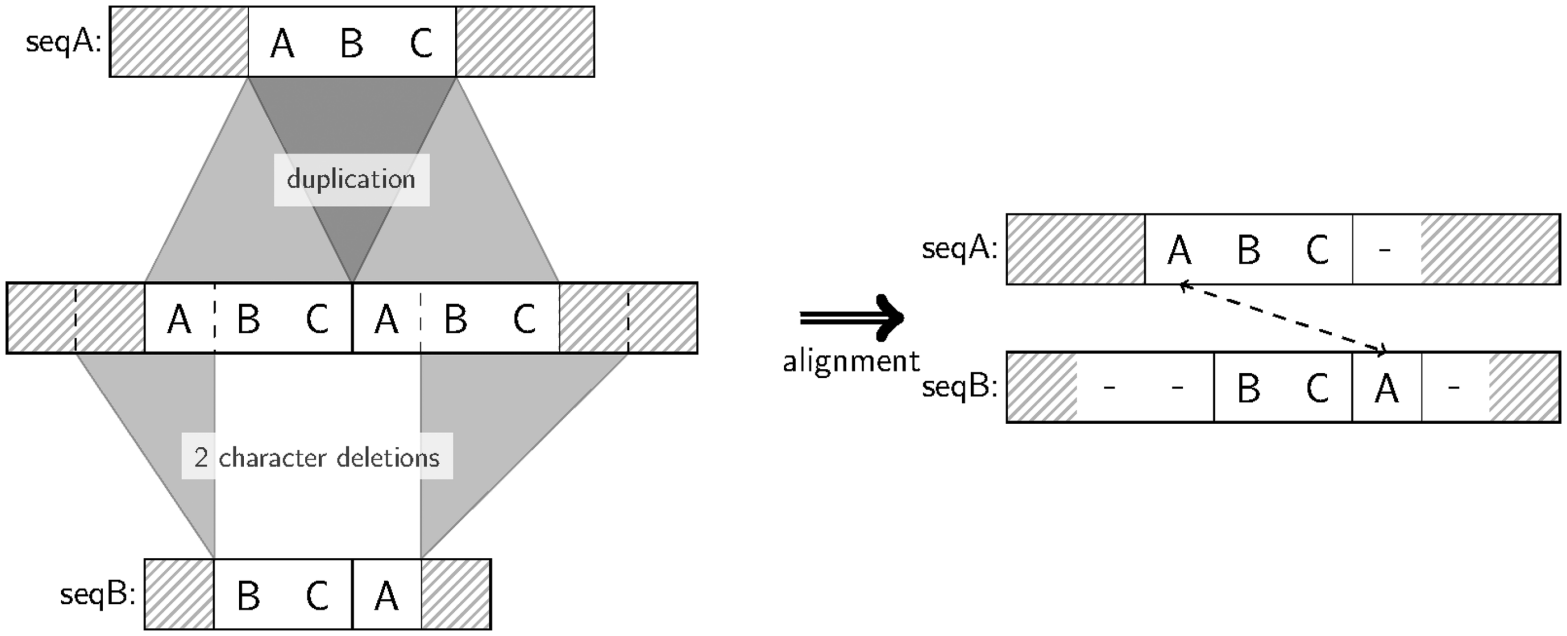
Not all alignable character pairs can be detected by global alignment. In the shown example, two homologous sequences seqA and seqB are separated by one TR unit duplication and two subsequent deletions. Each character in the TR region of seqA retains a corresponding homologous character in seqB, but the global alignment is unable to detect all such relationships owing to the requirement of retaining the character order of each sequence (character A in seqA is not aligned to the homologous A in seqB). Although such alignment does not fully reflect the homology in terms of aligned pairs, it nevertheless correctly reflects the three indels.

In addition, most current alignment methods for sequences with rearrangements and repeats (5,19) infer local alignments of all TR units, which are uninformative about the evolutionary events separating the whole sequences. To our knowledge, the only global MSA solution for sequences with repeats assumes fixed repeat unit boundaries (4), and, unfortunately, the respective implementation RAlign currently is not available. Here, we present a method to infer global MSAs of homologous sequences with TRs. A global alignment restricts the homology representation so that not all possible homologies can be identified owing to the requirement to retain the order of sequence characters (Figure 2): A global alignment informs about the sequence-level homology but cannot show the TR-level homology (i.e. between TR duplicate copies). However, the global alignment format is indispensable for many evolutionary analyses, including phylogenetic inference, positive selection, TR unit indel rate estimation and so forth.

We describe and implement a model of TR unit evolution, capitalizing on the advantages of a recent graph-based phylogeny-aware MSA algorithm (20). In our new algorithm, the TR unit indels are distinguished from normal character indels. Therefore, unlike any previous tools, our implementation ProGraphMSA+TR enables the rate estimation for TR unit indels. This helps to disentangle TR units and to measure indel events in a biologically meaningful way, improving the overall quality of reconstructed global alignments. This work paves the way for further analyses of this important category of proteins and their evolutionary properties. We model the TR evolution by incorporating special insertions and deletions spanning whole multiple TR units into the common evolutionary model of character substitutions, insertions and deletions. The new method is thus not constrained to duplication events and should be able to detect all changes in TR regions due to recombination, strand slippage and other events which insert or delete one or multiple TR units.

ProGraphMSA+TR inherits all other advantages of our previous graph-based alignment method (20), including context-specific profiles (21) and phylogeny-aware penalization of indels (22), and adds an affine cost model for TR unit indels. Information about putative TR regions is obtained automatically by transparent calls to third-party TR detectors such as T-REKS (23) or TRUST (24). Additional TR prediction software can be integrated with wrapper scripts.

We evaluate the new algorithm in a simulation framework incorporating TR evolution, by either sampling TR units from a profile hidden Markov model (HMM) or by mimicking strand replication slippage with duplications.

## MATERIALS AND METHODS

The new TR indel method has been implemented within ProGraphMSA (20), a framework for global progressive alignment with a graph-based representation of ancestral sequences. The algorithm starts by aligning linear graphs representing leaf sequences in a pairwise fashion as indicated by a guide tree. In further steps, these graphs are coalesced to directed acyclic graphs at the internal nodes of the guide tree—the paths through the graph representing possible ancestral sequences. The edges of the sequence graphs encode the indel history during the alignment. The alignment of two directed acyclic graphs boils down to the selection of homologous paths in both graphs. Although classic progressive alignment penalizes insertions multiple times (22), this method allows differentiating insertions from deletions by selecting the corresponding paths through the graph and thus penalizes them correctly.

### Modeling TR unit indels

TR unit indels were modeled by paths in the graph that bypass whole repeat units. This is achieved by adding edges to the leaf sequences to allow for skipping one or multiple repeat units. The edge penalties are adjusted to account for unit indel costs. Although theoretically, an arbitrary cost model is possible, we used affine gap penalties. Also for TR unit indels, the graph-based approach is able to differentiate insertions from deletions and thus penalizes them correctly.

**Figure 3.**
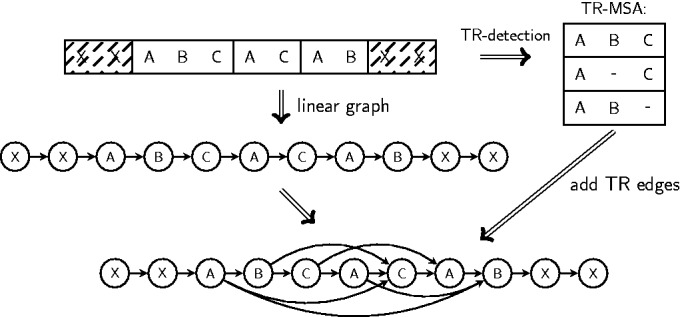
Possible TR unit indels are inferred from a TR-MSA. This TR-MSA is obtained transparently by running a TR detection program. For each character in the TR region, edges ending at the character after a TR-homologous character are added to the sequence graph.

To annotate the TR regions in a sequence with additional edges, ProGraphMSA+TR requires an MSA of homologous TR units (TR-MSA) for each type of TR in the given sequence. For each sequence to be aligned, ProGraphMSA+TR transparently calls TR detector programs T-REKS (23) or TRUST (24) to obtain TR predictions and TR unit alignments (Figure 3). For ancestral graphs at internal guide tree nodes, the TR information from all descendant leaves is incorporated. Each type of TR is processed separately, and edges are added to the sequence graph to allow for skipping one or multiple TR units at each possible position inside the TR region. To enable skipping of whole TR units, the target character of an edge has to follow a character that is TR-level homologous to the source character of the edge. When the homologous position is not present (e.g. as a result of a deletion), the target becomes the character following the virtual position of the absent homologous character (Figure 3). If during alignment, a TR edge is selected to be a part of the aligned paths, it is permanently added to the graph and henceforth represents an alternative path in the ancestral sequence due to an insertion or deletion.

Intuitively, our method should offer the best performance in reconstructing the evolution of TRs in homologs of moderate divergence and while the TR unit indel rate is low enough to produce non-overlapping events. Our approach allows only for TR unit indel events and does not enforce the alignment to be consistent in terms of TR-level homologies, i.e. two TR-level homologous characters in a sequence can be aligned to two non-homologous characters in the second sequence. Although such consistency could be achieved by column-wise alignment [e.g. profile-profile alignment (25)] of the TR unit MSAs of the two sequences, we chose not to enforce this consistency by default owing to possible errors in the TR unit alignments provided by TR detectors.

### Simulation of proteins with TR regions

**Figure 4.**
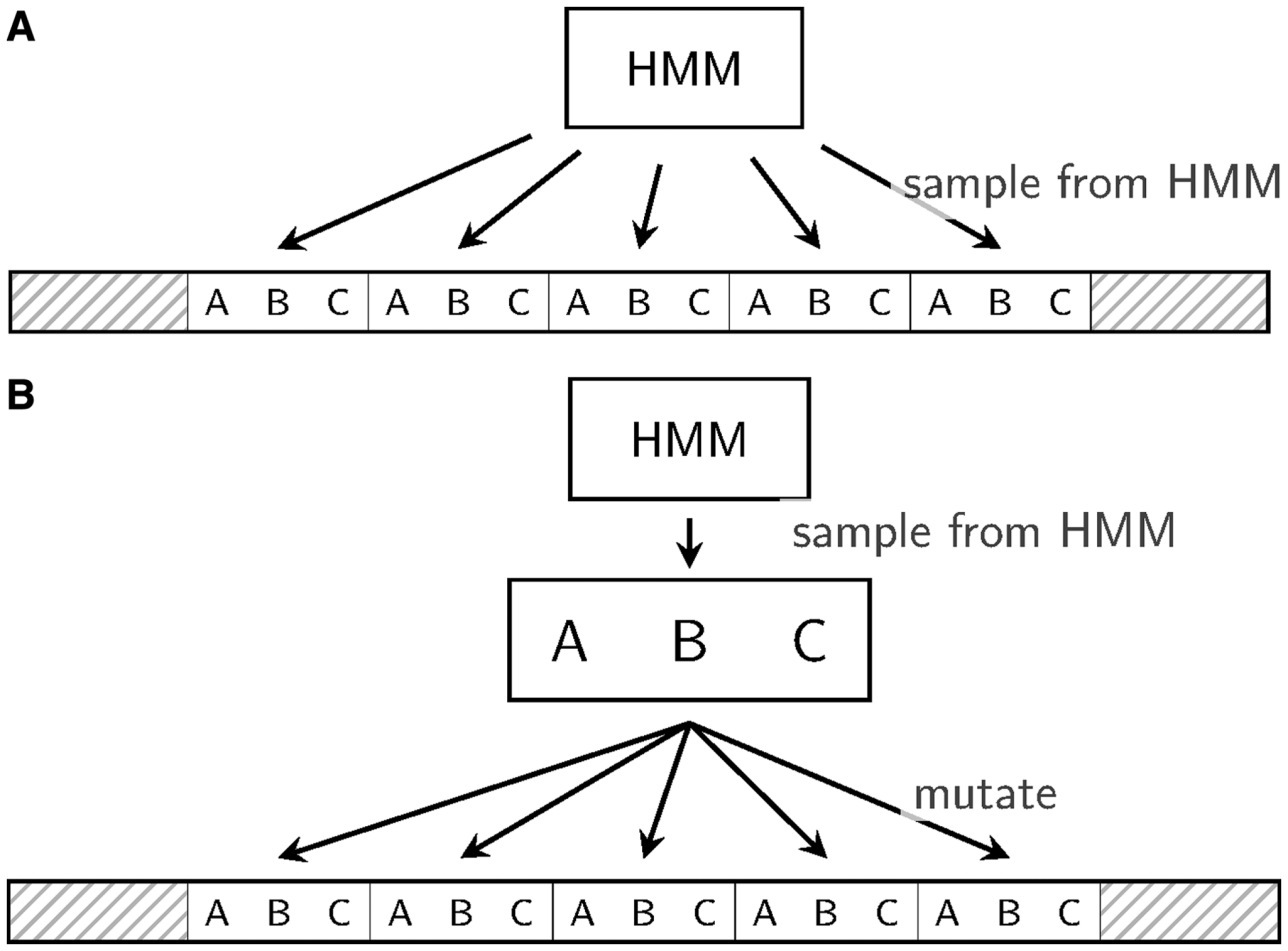
Simulated sequences consist of flanking regions of variable length (∼100 aa) and 6–20 TR units. These TR units are either sampled directly from a profile HMM (profile version) or a single unit is sampled and mutated at distance 0.2 expected substitutions per site (duplication version).

To evaluate ProGraphMSA+TR, we designed a simulation algorithm, which evolved an ancestral sequence containing TRs into a set of extant sequences. The mutation process was modeled using the WAG substitution model (26) with character insertions and deletions along six-taxa trees randomly sampled from birth–death models for speciation and extinction (27). Ten thousand MSAs with TRs were simulated for each of the total 60 parameter combinations (summarized in Table 1) and for both simulation scenarios (Figure 4). Each simulated ancestral sequence included two random flanking regions at the terminals and a core of 6 to 20 TR units (Figure 4) generated from their corresponding profile HMM (28). To allow for different functional and structural constraints of TRs as well as unit lengths, we simulated TRs representing different structural categories (29). We thus constructed four profile HMMs from TRs of the anti-freeze protein (AFP), GALA leucine rich repeats (LRR), zinc finger domain (ZNF) and spectrin repeats (SPT). These examples not only represent TRs with a wide range of unit length and numbers but also correspond to some of the most abundant and well-studied cases of TRs. AFPs facilitate organism’s survival at extremely low temperatures. APFs are remarkably diverse and widely found in vertebrates, fungi, plants and bacteria. LRRs are another diverse and frequent group of protein TRs. They are intensively studied because of their crucial role in the formation of protein–protein interactions. The most abundant known TR is probably the zinc finger motif, which forms zinc-coordinating 3D structures. ZNFs play versatile binding roles and thus facilitate a great variety of protein functions from translation and transcription to more specialized functions such as chromatin remodeling and cytoskeleton organization (30). Finally, SPT repeats are an interesting example of long TRs that nevertheless exhibit large unit numbers. SPTs play role in a number of proteins involved in cytoskeletal structure.

**Table 1.** Simulation parameters

Insertion rate	0.005 (per site)
Deletion rate	0.005 (per site)
Indel length distribution	geometrical (mean = 3.5)
Substitution model	WAG
Tree taxa in sample	6
Tree topology	constant rate birth-death model
Tree lengths	0.1 0.5 1.0
Flanking region length	geometrical (mean = 100)
TR unit type	AFP, LRR, ZNF, SPT
TR unit length	10, 24, 24, 100
Ancestral TR units	20, 6, 6, 10
TR insertion rates	0.1 0.5 1 2 4 (per unit)
TR deletion rates	0.1 0.5 1 2 4 (per unit)

(i) Simulation from the profile HMM:	
TR indel length distribution	geometrical (mean = 1.5 units)

(ii) Simulation by TR unit duplication:	
TR indel length distribution	empirical (mean = 1.1 − 3.7 units)

Two simulation methods were used to generate protein sequences with TRs. (i) Simulation from the profile HMM: Each TR unit in a sequence was sampled from a profile HMM and (ii) Simulation by TR unit duplication: one TR unit is sampled from a profile HMM but the consequent TR units are generated by duplicating a part of the existing TR sequence. AFP = AFP repeat (PF02420); LRR = GALA LRR (31,32); ZNF = zink finger domain (PF00096); SPT = spectrin repeat (PF00435).

Simulated TRs had average unit lengths between 10 and 100 amino acids. In the core TR region, we additionally simulated insertions and deletions of whole TR units with different insertion/deletion rates and different length distributions (one or multiple units at once). Most importantly two distinct modes were used for simulating TR unit indel events.

‘The profile method’ sampled all inserted TR units (including the ancestral units) from a profile HMM (Figure 4a). As unit insertions were allowed to occur at each position inside the TR region, a randomly sampled TR unit insertion was ‘rotated’ to a suitable phase before the insertion. Deletions were performed similarly on whole TR units, i.e. starting at a random position inside the TR region and up to a subsequent TR-level homologous character according to the assumed length distribution. When a corresponding TR-level homologous character was missing (due to a deletion), the profile-based simulation still performed a deletion of the given number of units up to the next position following the missing character homolog (Figure 3).

‘The duplication method’ mimicked strand slippage mutations (33) by duplicating or deleting parts of the TR region between two randomly chosen TR-level homologous characters. The required number of ancestral TR units is evolved from a single copy on a star topology and then concatenated to form the TR region (Figure 4b).

All simulation parameters were identical for the two methods and the four TR types, except for the ancestral unit counts and the length distributions of TR unit indels (Table 1). Although the profile method used the geometric distribution to determine the number of inserted/deleted units per event, the TR unit duplication method exponentially weighted each possible pair of TR-homologous sequence characters by the length of the inserted/deleted segment. Based on these weights, a pair was chosen randomly and the region in between either duplicated or deleted. Our weighting scheme resulted in the average TR indel length 1.1–3.7 units per indel, depending on the TR unit length and the number of units, which was influenced by the tree length and the TR unit indel rate.

## RESULTS AND DISCUSSION

The comparative performance of different methods was evaluated on simulated data generated with different TR unit types, different mutation and indel rates, and for two distinct simulation modes of TR unit indels (Table 1). To assess the advantage of our TR-aware alignment algorithm, ProGraphMSA+TR was executed either with no prior knowledge of TR units, with true TR units as known from the simulation, or with TR information reconstructed by the TR predictor TRUST (24). The performance has been measured with regard to (i) the number of correctly aligned character pairs as compared with the true reference alignment and (ii) the number of inferred TR unit indels (as one of the goals of the method was the inference of TR unit evolution).

### Performance assessment based on pairwise measures

To evaluate the inferred MSAs in terms of correctly aligned residue pairs, we used two common alignment-quality statistics fD and fM. The ‘developer score’ (fD) is the fraction of correct pairs relative to the total number of pairs in the reference alignment, i.e. it is a measure of ‘sensitivity’ (also known as power). The ‘modeler score’ (fM) is computed relative to the number of pairs in the reconstructed MSA and thus provides a measure of *specificity* (or accuracy).

For profile-based simulation of protein alignments with TRs, the definition of fD and fM is straightforward as TR unit insertions do not create ambiguous pairings. However, in the presence of TR unit duplication events, a global MSA becomes ambiguous: each copy of a unit can be treated either as homologous or as an insertion, i.e. every character of the corresponding TR region in the ancestral sequence has two (or more) possible pairings in the alignment, but only one can be realized owing to the nature of global alignments.

**Figure 5.**
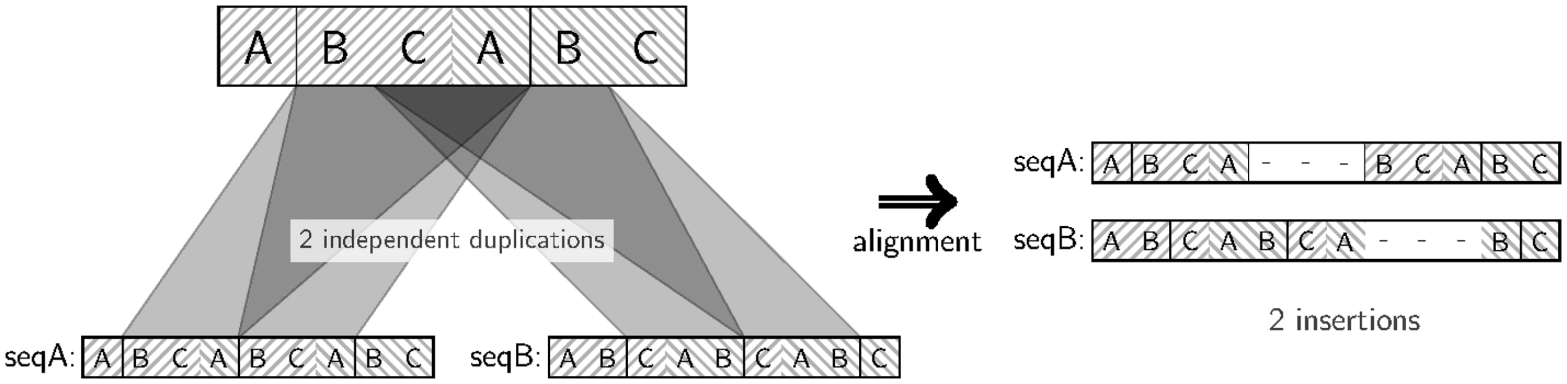
Only one aligned pair is allowed per ancestral character. Consider the central characters C and A, which are independently duplicated in both leaf sequences. By allowing only one aligned pair per ancestral character, the number of indel events is reconstructed correctly.

Consider a scenario in which an ancestral TR unit was duplicated independently in disjoint clades (Figure 5). Each character in the TR unit of one leaf sequence has two possible homologous pairings with characters in the other leaf sequence, i.e. a total of four possible pairings for a single ancestral character. As the aim is to estimate the correct number of insertions rather than reconstruct all possible pairwise homologies, only one of four possible pairs can be assumed correct. Moreover, there exist scenarios in which not all possible pairings can be realized in the alignment owing to the representation of global alignments (Figure 2). Thus, the maximum achievable sensitivity is not always 1. However, and more importantly, in contrast to local alignments, the number of inferred TR unit indels will be inferred correctly.

The quality of the inferred MSAs was assessed separately for TR regions and their flanking sequences. Table 2 summarizes the command line parameters used to run the alignment programs. The inferred MSAs were compared in terms of the fD and fM statistics for ProGraphMSA (without TR information), ProGraphMSA+TR and the popular programs MUSCLE (34) and MAFFT (35).

**Table 2.** Command line parameters

Program (version)	Parameters	Description
ProGraphMSA	-i 2 –mldist	Iterate distance and guide tree estimation two times
	–no_force_align –end_indel_prob -1	No special treatment of terminal gaps
	–fasta -o prograph.fasta input.fasta	Input/output

ProGraphMSA+TR	–repeats –custom_tr_cmd trust2treks.py	Use TRUST for TR detection
	–repeat_indel_rate 0.1	TR unit insertion/deletion rate (per site)
	–repeat_indel_ext 0.3	TR units insertion/deletion length coefficient
	-i 2 –mldist	Iterate distance and guide tree estimation two times
	–no_force_align –end_indel_prob -1	No special treatment of terminal gaps
	–fasta -o prographTR.fasta input.fasta	Input/output

ProGraphMSA+realTR	–read_repeats repeats.treks	Use true repeat information
	–repeat_indel_rate 0.1	TR unit insertion/deletion rate (per site)
	–repeat_indel_ext 0.3	TR units insertion/deletion length coefficient
	-i 2 –mldist	Iterate distance and guide tree estimation two times
	–no_force_align –end_indel_prob -1	No special treatment of terminal gaps
	–fasta -o prographRealTR.fasta input.fasta	Input/output

MAFFT 6.843-with-ext	–maxiterate 1000 –globalpair	Iterative refinement (suggested for best results)
	–quiet input.fasta > mafft.fasta	Input/output

MUSCLE 3.8.31	-quiet -in input.fasta -out muscle.fasta	Input/output

All versions of ProGraphMSA have common parameters to iterate the estimation of distances and guide tree and to disable the special treatment of terminal gaps, which are enabled by default to deal with real sequence data. Further, the TR versions use the same TR unit indel distributions but use different sources for TR information. ProGraph+TR uses a TR-MSA produced by TRUST, whereas ProGraph+realTR uses the true TR unit information provided by the simulation algorithm. MUSCLE was executed with the default parameters and MAFFT with the parameters that were suggested in the command line help for best results.

As the observed performance was similar for all types of TR units, full results are shown only for LRR-containing MSAs (Figures 6 and 7). The results for the MSAs containing AFP, ZNF and SPT are provided as supplementary material (Supplementary Figures S1–S6).

**Figure 6.**
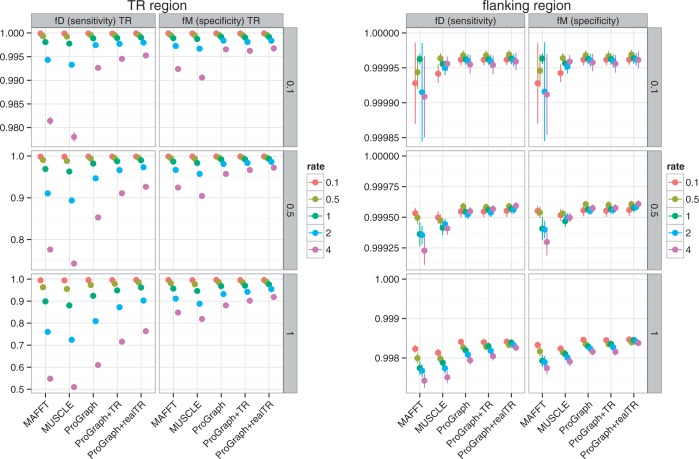
Results for the profile simulation method simulation of MSAs with GALA-LRR-like repeats. The vertical facets represent sequence divergence (i.e. total tree lengths measured in expected substitutions per site) used in simulation. ProGraphMSA was executed without any additional information on TRs, whereas ProGraphMSA+TR used TR information detected by TRUST, and ProGraphMSA+realTR was executed with the true TR-MSA provided (as known from simulation). Results for the popular programs MAFFT and MUSCLE are depicted for a qualitative comparison.

**Figure 7.**
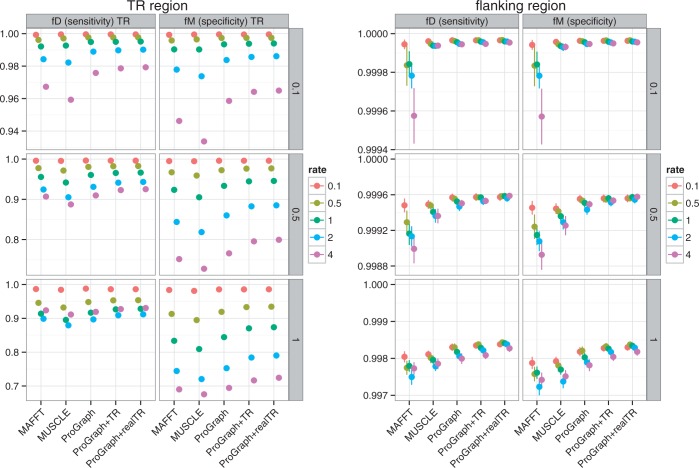
Results for the duplication method simulation of MSAs with GALA-LRR-like repeats. The vertical facets represent sequence divergence (i.e. total tree lengths measured in expected substitutions per site) used in simulation. ProGraphMSA was executed without any additional information on TRs, whereas ProGraphMSA+TR used TR information detected by TRUST, and ProGraphMSA+realTR was executed with the true TR-MSA provided (as known from simulation). Results for the popular programs MAFFT and MUSCLE are depicted for a qualitative comparison.

For programs that do not account for TR presence (ProGraphMSA, MAFFT and MUSCLE), we observed similar fD and fM values, which decreased, as expected, with increasing TR unit indel rates. In contrast, accounting for the presence of TRs significantly improved the performance: In general, we can observe that ProGraphMSA+TR outperformed ProGraphMSA—both in terms of sensitivity and specificity. Moreover, with true TRs (as known from simulation) ProGraphMSA+TR had better sensitivity and specificity compared with using the predicted TR units (compare ProGraphMSA+realTR versus ProGraphMSA+TR in Figures 6 and 7). Even though the TR prediction by TRUST is often far from perfect (14), it nevertheless aids to infer a more accurate MSA for sequences with TRs (Supplementary Figure S10). Interestingly, the awareness of TRs helps to improve the alignment of both the TR region and the flanking regions, even if not always significantly.

Overall, the simulation results for duplication and profile methods were similar, also when comparing data sets with different types of TR units. The differences in absolute values of fD and fM for the two simulation methods appear to be due to different lengths and divergences of simulated TR regions. Indeed, on average, the profile simulation method increased the number of TR units during evolution for longer trees and high TR unit indel rates, whereas the duplication simulation method decreased the total length of the TR region. Only in the case of low TR unit indel rates or short trees for data simulated with the profile method, we observed a significant but still small decrease in specificity or sensitivity of ProGraphMSA+TR compared with ProGraphMSA. Particularly, owing to the high divergence between TR units sampled from the profile HMM, the lower quality of TR predictions led to slightly lower specificity compared with ProGraphMSA without TR information. We similarly observe that the TR detection performs worse for short repeat units (Supplementary Figures S7–S9). Remarkably, the net performance advantage was always in favor of modeling TRs, as ProGraphMSA+TR achieved the best balance of combining sufficiently high specificity with clear gains in sensitivity.

### TR unit indel rate estimation

To our knowledge, the presented algorithm and its implementation is currently the only method that allows the detection of TR unit indels from sequence data during alignment. The rate of TR unit indels cannot be reliably inferred based only on TR unit counts in homologous sequences, as TR detection methods are not sufficiently accurate (14). In particular for distantly related TRs, they often fail to detect individual units or identify a different repeat unit size (see example in Supplementary Figure S10). Methods based on global alignment usually provide better power to infer indels, especially if they are able to discriminate between insertions and deletions in ancestral states during phylogenetically guided alignment (22,36).

However, TR-unaware MSA algorithms tend to merge TR unit indels if their cost model does not account for frequent and long indels. In addition, TR unit indels can be split if gaps of the correct unit length are not favored (compare examples in Supplementary Figure S11). Hence, these methods do not facilitate accurate inference of TR unit indel events. Conversely, ProGraphMSA+TR explicitly marks possible TR unit indels in its reconstructed ancestral sequences. Combined with an appropriate cost model our approach facilitates accurate inference of TR unit indels.

**Figure 8.**
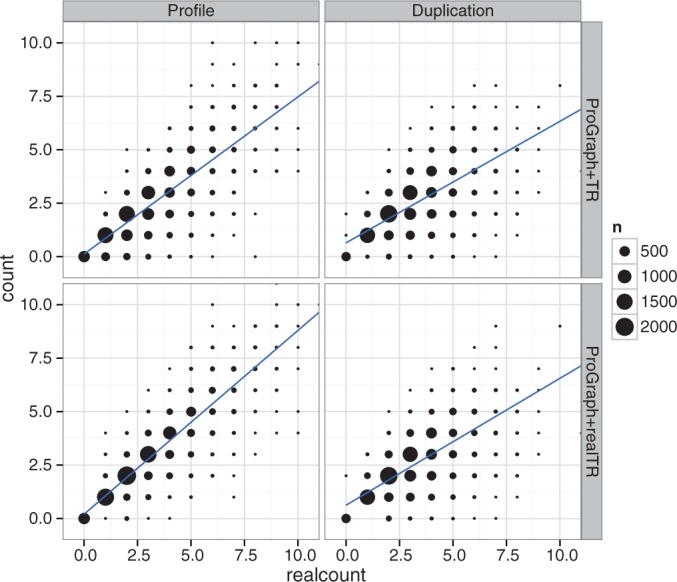
Real versus estimated number of TR unit indel events for MSAs with GALA-LRRs, tree length 0.5 and high TR unit indel rate of 1.0. As expected, the number of TR unit indel events was usually underestimated because of nested indels on single branches and multiple indels being erroneously merged.

We assessed the robustness of TR unit indel estimation by comparing the number of inferred TR indel events with the true numbers as known from the simulation. Overall, we observed a strong correlation of estimated and true indel counts. For example, Figure 8 shows the results of a linear regression for sequences with moderate TR unit indel rate (1.0 per unit) and moderate divergence (tree length = 0.5 expected substitutions per character) for MSAs with GALA LRRs. As expected, for high indel counts, the number of events was underestimated owing to overlapping indels and erroneous merging of close indels. Again, for the profile-based simulation, the TR detector TRUST (24) failed to detect several repeat units owing to higher divergence between the units emitted by the profile HMM. This trend is even stronger for short repeat units (Supplementary Figures S7–S9). Consequently, the number of detected TR unit indel events was significantly lower compared with when the true TR information was used.

We note that the results were affected by the choice of input parameters in ProGraphMSA+TR, namely, the TR unit indel rate and its extension probability. These parameters should be provided by the user; yet, they are rarely known. We suggest default values (Table 2); however, for more reliable TR unit rate estimation, we recommend to perform ProGraphMSA+TR in an iterative fashion, adjusting the parameters to newly estimated values until convergence. Indeed, our simulation experiment (Table 3) demonstrated that in practice, the indel rate parameter had only a small effect. One additional iteration was already sufficient to obtain an estimate of the correct order of magnitude. Using this estimate from the first analysis to re-estimate the TR unit indel rate yielded results, which were close to those obtained with the true value. We also observed some improvement in alignment quality related to a better choice of the input TR indel rate (results not shown).

**Table 3.** Effect of ProGraphMSA+TR’s unit indel rate parameter on TR rate estimation

	Initial	1 iteration	Real
Parameter	0.1	0.00779	0.0083
Error	0.081 ± 0.036	−0.053 ± 0.027	−0.070 ± 0.028
Abs. error	0.225 ± 0.034	0.135 ± 0.025	0.138 ± 0.027

Parameter	0.1	0.342	0.417
Error	1.60 ± 0.11	1.42 ± 0.11	1.38 ± 0.11
Abs. error	1.67 ± 0.11	1.59 ± 0.10	1.54 ± 0.10

In the upper half of the table, the indel rate was chosen to be lower than ProGraph+TR’s default parameter. With an indel rate of 0.2 per unit and a unit length of 24 (GALA-LRR), the ideal setting for the parameter would be 0.0083. After one iteration with the wrong parameter, we estimated the rate parameter to 0.00779, which was already close to the true value and also yielded results, which were similar to those obtained with the true parameter value. The reported error and absolute error are in terms of TR unit indel counts and should be contrasted to the expected number of unit indels, which is 2 under the simulated tree length and unit count. In the lower half of the table, we used a unit indel rate of 10 and therefore expected on average 10 unit indels per tree. Again, after one iteration, the parameter was estimated sufficiently close to the true value.

The input TR unit indel rate is the indel rate per TR site. To obtain a comparable parameter value from ProGraphMSA+TR, the inferred TR unit indel rate has to be divided by the estimated length of the TR region.

### Applications to bacterial GALA proteins with LRR

To illustrate the advantages of ProGraphMSA+TR, we analyzed TR unit evolution in the GALA proteins with LRRs arranged in tandem—from the phytopathogenic bacteria *Ralstonia solanacearum*. These proteins are a family of type III effectors—the major pathogenicity determinant of the *R*. *solanacearum* species complex (37,38). Each LRR forms a coil, and together in tandem, GALA-LRR regions fold in a horse-shoe shaped structure, which was proposed to have a key functional role: the convex surface of each LRR structure is hypothesized to be a binding site important for GALA’s adaptor function (31). Consequently, variation in LRR numbers and the indel process governing LRRs is of relevance for studies of the functional diversification of different GALA subfamilies and their differential virulence properties.

**Figure 9.**
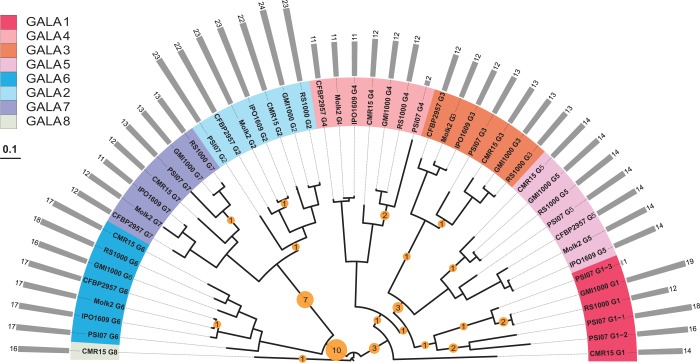
The evolution of LRR tandem units in GALA proteins from *Ralstonia solanacearum*. Yellow circles represent the numbers of LRR indels inferred by ProGraphMSA+TR and are mapped to the corresponding nodes of the GALA phylogeny inferred by Remigi *et al.* (2011). Colored taxonomic ranges represent different paralogous GALA families. Numbers of LRR units in each strain are represented by gray columns.

We analyzed the GALA paralogs from two different studies (31,32). ProGraphMSA+TR was applied to re-align different GALA data sets and to infer estimates of TR unit indel counts. These numbers and rates are summarized in Table 4. Such analyses usually require known tree root position. In an absence of such, the problem may require an additional optimization for the location of the root based on minimizing the total TR indel number on the phylogeny relating the homologous set under consideration. Inferred indel event counts were mapped on the GALA phylogeny (32) and are depicted in Figure 9 together with the distribution of numbers of LRR units in GALA paralogs. The numbers of TR units were detected by a profile HMM as implemented in HMMER (39) based on the GALA-LRR profile. Although LRR numbers varied substantially between the different paralogs, we observed strong conservation of LRR unit numbers within all individual GALA clades with an exception of GALA1. Consequently, the TR indel rate in GALA1 was the highest with 0.43 of expected TR indels per unit. In contrast, TR indel rates were particularly low in GALAs 2, 4 and 5, suggesting that the preservation of LRR numbers may be relevant to the preservation of advantageous properties that different GALAs have evolved. The number of LRRs in different paralogs may be relevant to their pathogenicity properties in different hosts, particularly owing to the hypothesized role of LRR regions to be involved in protein binding. This is consistent with experimental studies that show that pathogenesis on a specific host plant is defined by the differential GALA requirement. However, the clear phylogenetic division of GALA paralogs on two subfamilies 2, 6, 7 and 1, 3, 4, 5 does not imply homogeneous forces acting on each subfamily. Rather the diversity within each subclade may play a specific role. For example, Remigi *et al.* (32) suggested that some degree of recombination coupled with selection operate on GALA 3, 4, 6 and 7. However, neither selection nor recombination can be detected on GALA 1, 2 and 5. Likewise, we do not observe homogeneity of TR indel rates within each of these two subclades.

**Table 4.** TR unit indel counts and rates in GALA LRRs

Data set	TR unit indels	Total branch length	Average TR units	Rate per unit	Rate per site
Kajava *et al.* (31)					
GALA1345	13	2.8140	15.1428	0.3051	0.0127
GALA267	10	2.4650	25.6667	0.1581	0.00659
All	31	4.3998	20	0.3523	0.0147

Remigi *et al.* (32)					
GALA1	12	2.077	13.3333	0.4333	0.01805
GALA2	2	1.5396	22.7143	0.05719	0.002383
GALA3	6	1.7045	10.7778	0.3266	0.01361
GALA4	1	1.6633	10.5	0.05726	0.002386
GALA5	1	1.5133	14	0.0472	0.001967
GALA6	3	1.6230	13.7778	0.1342	0.00559
GALA7	2	1.8470	12.2222	0.0886	0.003691
All	41	11.3260	14.6531	0.2470	0.01029

Results for the data sets from Kajava *et al.* and Remigi *et al.* The rate per unit is computed from the number of TR unit indels divided by the total branch length of the gene tree and the average number of TR units. The rate per site is just the rate per unit divided by 24, the unit length.

Overall, our analysis clearly supports that GALA proteins evolve through diversification, possibly using LRR number variation (in combination with positive diversifying selection on the protein) to create a specific repertoire contributing to pathogenesis on different plant hosts.

The TR indel process acting on the GALA family appears to be a characteristic force creating and maintaining the allelic variability.

## CONCLUSION

We presented a fast and accurate method for global alignment of multiple sequences with TRs. In contrast to local alignment methods, our method is able to detect insertions and deletions of TR units which are not restricted to TR unit boundaries. The method is implemented in ProGraphMSA+TR, which is built on a graph-based alignment framework (20), and thus inherits not only its speed but also robustness to errors in the guide tree or presence of alternative splicing variants and other features like context-specific profiles (21) and phylogeny-aware penalization of insertions (22).

In this study, ProGraphMSA+TR has been shown to improve the quality of alignments by incorporating TR information, which can be obtained using one of many available TR detection methods, or a combination of different TR detectors and a subsequent statistical scoring of putative TR units (14). More accurate information about TR units in sequences leads to better quality of estimated MSAs. Compared with other alignment programs, the advantage of ProGraphMSA+TR is stronger for more divergent sequences or with high TR indel rates.

In addition to improved alignment quality, the new method allows for an accurate inference of TR unit indel events, which facilitates the estimation of TR indel rates. This is enabled by placing indels in a phylogenetically meaningful fashion (22) as well as distinctive modeling of TR unit indels in contrast to traditional non-TR events. Methods that do not specifically account for TR unit indels will eventually merge or split TR-related events thus depriving subsequent analyses of an obvious way to disentangle TR-related evolutionary events.

Here, we demonstrated the utility of ProGraphMSA+TR to study the evolution and diversification of paralogous TR regions on real data, the GALA proteins with LRRs that act as type III effectors in phytopathogenic bacteria *R*. *solanacearum*. Such studies may provide additional insight for detecting selective advantages from phylogenetic patterns of TR indel rates and evolutionary constraints on TR unit numbers.

Given the high abundance of TRs in genomic sequences (14), modeling TR indels becomes important to improve alignments of DNA or protein sequences and to further study TR evolution and their contribution to functional changes. Even with imperfect prediction of TRs, our method performs at least as well as best-performing aligners, with clear advantages when TR mutation and indel rates are high. Overall, for easy cases (where most aligners perform well), our method performs at least as well as the top-performing aligners, and often with a small improvement of 1–2%. For difficult cases (high TR rates, divergence), our new approach can offer a respectable improvement of 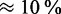. Thus, we can recommend ProGraphMSA+TR to anyone seeking high-quality global MSAs for protein or DNA sequences. For convenience of user community, a web server for ProGraphMSA+TR is provided.

Finally, our models for simulating sequences with TRs and their evolution allow for the most realistic to date simulation scenarios of such sequences. The implementation of the simulation algorithm is available from the project website (later in the text) and should be a useful standalone tool for other studies of TRs and their evolution.

## AVAILABILITY

Both the implementation of TR-aware sequence alignment and the simulation algorithm are available from the project website.

- Project name: ProGraphMSA+TR
- Project home page: http://sf.net/projects/prographmsa/
- Operating system: Platform independent
- Programming language: C++
- Other requirements: Eigen 3.1, TCLAP 1.1 or higher
- License: GNU GPLv3
- Web server: http://www.cbrg.ethz.ch/services/ProGraphMSA

## SUPPLEMENTARY DATA

Supplementary Data are available at NAR Online.

## FUNDING

Funding for open access charge: Swiss National Science Foundation [ref. 31003A-127325 to M.A.] and the Germaine de Staël program of the Swiss Academy of Engineering Sciences [ref. 2011-15]; ETH Zürich (to A.M.S. and M.A.).

*Conflict of interest statement*. None declared.

## Supplementary Material

Supplementary Data

## References

[1] Löytynoja A, Goldman N (2008). Phylogeny-aware gap placement prevents errors in sequence alignment and evolutionary analysis. Science.

[2] Löytynoja A, Vilella AJ, Goldman N (2012). Accurate extension of multiple sequence alignments using a phylogeny-aware graph algorithm. Bioinformatics.

[3] Anisimova M, Cannarozzi G, Liberles DA (2010). Finding the balance between the mathematical and biological optima in multiple sequence alignment. Trends Evol. Biol..

[4] Sammeth M, Heringa J (2006). Global multiple-sequence alignment with repeats. Proteins.

[5] Phuong TM, Do CB, Edgar RC, Batzoglou S (2006). Multiple alignment of protein sequences with repeats and rearrangements. Nucleic Acids Res..

[6] Ledergerber C, Dessimoz C (2007). Alignments with non-overlapping moves, inversions and tandem duplications in o(n 4) time. Computing and Combinatorics Lecture Notes in Computer Science.

[7] Sammeth M, Weniger T, Harmsen D, Stoye J (2005). Alignment of tandem repeats with excision, duplication, substitution and indels (EDSI). Algorithms in Bioinformatics.

[8] Treangen TJ, Darling AE, Achaz G, Ragan MA, Messeguer X, Rocha EPC (2009). A novel heuristic for local multiple alignment of interspersed DNA repeats. IEEE/ACM Trans. Comput. Biol. Bioinformatics.

[9] Blanco E, Guig R, Messeguer X (2007). Multiple non-collinear TF-map alignments of promoter regions. BMC Bioinformatics.

[10] Usdin K (2008). The biological effects of simple tandem repeats: lessons from the repeat expansion diseases. Genome Res.

[11] Wyman AR, White R (1980). A highly polymorphic locus in human DNA. Proc. Natl Acad. Sci..

[12] Jeffreys AJ, Wilson V, Thein SL (1985). Individual-specific’fingerprints’ of human DNA. Nature.

[13] Marcotte EM, Pellegrini M, Yeates TO, Eisenberg D (1999). A census of protein repeats. J. Mol. Biol..

[14] Schaper E, Kajava AV, Hauser A, Anisimova M (2012). Repeat or not repeat? Statistical validation of tandem repeat prediction in genomic sequences. Nucleic Acids Res..

[15] Szalkowski AM, Anisimova M (2011). Markov models of amino acid substitution to study proteins with intrinsically disordered regions. PLoS One.

[16] Levinson G, Gutman GA (1987). Slipped-strand mispairing: a major mechanism for DNA sequence evolution. Mol. Biol. Evol..

[17] Ellegren H (2000). Microsatellite mutations in the germline:: implications for evolutionary inference. Trends Genet..

[18] Mirkin SM (2006). DNA structures, repeat expansions and human hereditary disorders. Curr. Opin. Struct. Biol..

[19] Raphael B, Zhi D, Tang H, Pevzner P (2004). A novel method for multiple alignment of sequences with repeated and shuffled elements. Genome Res..

[20] Szalkowski A (2012). Fast and robust multiple sequence alignment with phylogeny-aware gap placement. BMC Bioinformatics.

[21] Biegert A, Söding J (2009). Sequence context-specific profiles for homology searching. Proc. Natl Acad. Sci..

[22] Löytynoja A, Goldman N (2005). An algorithm for progressive multiple alignment of sequences with insertions. Proc. Natl Acad. Sci. USA.

[23] Jorda J, Kajava AV (2009). T-REKS: identification of tandem REpeats in sequences with a k-meanS based algorithm. Bioinformatics.

[24] Szklarczyk R, Heringa J (2004). Tracking repeats using significance and transitivity. Bioinformatics.

[25] Edgar RC, Sjölander K (2004). A comparison of scoring functions for protein sequence profile alignment. Bioinformatics.

[26] Whelan S, Goldman N (2001). A general empirical model of protein evolution derived from multiple protein families using a maximum-likelihood approach. Mol. Biol. Evol..

[27] Gernhard T (2008). The conditioned reconstructed process. J. Theor. Biol..

[28] Eddy SR (1998). Profile hidden markov models. Bioinformatics.

[29] Kajava AV (2012). Tandem repeats in proteins: from sequence to structure. J. Struct. Biol..

[30] Laity JH, Lee BM, Wright PE (2001). Zinc finger proteins: new insights into structural and functional diversity. Curr. Opin. Struct. Biol..

[31] Kajava AV, Anisimova M, Peeters N (2008). Origin and evolution of GALA-LRR, a new member of the CC-LRR subfamily: from plants to bacteria?. PLoS One.

[32] Remigi P, Anisimova M, Guidot A, Genin S, Peeters N (2011). Functional diversification of the GALA type III effector family contributes to ralstonia solanacearum adaptation on different plant hosts. New Phytol..

[33] Jorda J, Xue B, Uversky VN, Kajava AV (2010). Protein tandem repeats - the more perfect, the less structured. FEBS J..

[34] Edgar RC (2004). MUSCLE: multiple sequence alignment with high accuracy and high throughput. Nucleic Acids Res..

[35] Katoh K, Misawa K, Kuma KI, Miyata T (2002). MAFFT: a novel method for rapid multiple sequence alignment based on fast fourier transform. Nucleic Acids Res..

[36] Westesson O, Lunter G, Paten B, Holmes I April 2012 accurate reconstruction of insertion-deletion histories by statistical phylogenetics. PLoS One.

[37] Angot A, Peeters N, Lechner E, Vailleau F, Baud C, Gentzbittel L, Sartorel E, Genschik P, Boucher C, Genin S (2006). Ralstonia solanacearum requires f-box-like domain-containing type III effectors to promote disease on several host plants. Proc. Natl Acad. Sci..

[38] Cunnac S, Occhialini A, Barberis P, Boucher C, Genin S (2004). Inventory and functional analysis of the large hrp regulon in ralstonia solanacearum: identification of novel effector proteins translocated to plant host cells through the type III secretion system. Mol. Microbiol..

[39] Eddy SR (2011). Accelerated profile HMM searches. PLoS Comput. Biol..

