# Supplementary material for: Graph-based modeling of tandem repeats improves global multiple sequence alignment
Source: Nucleic Acids Res. 2013 Jul 22;41(17):e162. doi: 10.1093/nar/gkt628 (PMC3783189; doi:10.1093/nar/gkt628)
Supplement: Supplementary Data [file supp_41_17_e162__index.html]

Graph-based modeling of tandem repeats improves global multiple sequence alignment — Graph-based modeling of tandem repeats improves global multiple sequence alignment — Supplementary Data 

# Graph-based modeling of tandem repeats improves global multiple sequence alignment

## 

files

**Files in this Data Supplement:**

- Supplementary Data - eps file
